# How much of a face is a face: exploring reidentification potential with generative AI

**DOI:** 10.1117/1.JMI.13.S1.S11202

**Published:** 2026-02-15

**Authors:** Chloe Cho, Yihao Liu, Bohan Jiang, Andrew J. McNeil, Benoit M. Dawant, Bennett A. Landman, Eric R. Tkaczyk

**Affiliations:** a Vanderbilt University, Department of Biomedical Engineering, Nashville, Tennessee, United States; b Vanderbilt University School of Medicine, Vanderbilt Medical Scientist Training Program, Nashville, Tennessee, United States; c Vanderbilt University, Department of Electrical and Computer Engineering, Nashville, Tennessee, United States; d Vanderbilt University Medical Center, Department of Dermatology, Nashville, Tennessee, United States; e Tennessee Valley Healthcare System, Department of Veterans Affairs, Dermatology Service and Research Service, Nashville, Tennessee, United States; f Vanderbilt University, Department of Radiology and Radiological Sciences, Nashville, Tennessee, United States; g Vanderbilt University, Department of Biomedical Informatics, Nashville, Tennessee, United States; h Vanderbilt University, Department of Computer Science, Nashville, Tennessee, United States

**Keywords:** generative AI, reidentification, facial images, privacy, deidentification, latent diffusion model

## Abstract

**Purpose:**

Clinical photographs play an integral role across medical fields. Since the mid-20th century, deidentification has consisted of black bars covering specific facial features, typically the eyes alone. Although increasingly questioned, this practice persists in clinical and academic settings.

**Approach:**

A barrier to standardized deidentification guideline development is the unknown risk of artificial intelligence (AI) to reconstruct faces from partially obscured photos. We evaluate the ability of generative AI to reconstruct 10,000 facial images in the Synthetic Faces High Quality dataset across 14 regional masking strategies.

**Results:**

Covering the eyes or any other single facial feature resulted in highly identifiable reconstructions, demonstrated by low face mesh distortion (0.14 to 0.18 relative to whole-face masking; absolute total face mesh distortion 8.34 to 10.19) and high structural similarity index to the original face (1.24 to 1.25 relative to whole-face masking; absolute SSIM 0.91 to 0.92). An open-source face verification model using Dlib was able to match 97.98% to 99.93% of these reconstructed images with the original image prior to single feature masking. Removing all major facial features (eyebrows, eyes, nose, and mouth) resulted in a threefold reduction in face verification rates compared with eyes alone, from 98.87% (95% CI [98.63%, 99.07%]) to 33.93% (95% CI [32.95%, 34.94%]).

**Conclusions:**

We provide quantitative metrics of the reidentification risk that modern generative AI technology poses for partially obscured facial images.

## 1 Introduction

Clinical photographs play an integral role across medical fields. To protect patient privacy, facial images are often deidentified by placing black bars over the eyes—a long-standing practice dating back to the mid-20th century that appears to obscure the most identifiable part of the face.^1^^,^^2^ Although increasingly questioned, this practice persists in clinical and academic settings amid a lack of standardized deidentification guidelines.^2^^–^^4^ A barrier to standardized deidentification guideline development is the unknown risk of artificial intelligence (AI) to reconstruct faces from partially obscured photos. Advancements in artificial intelligence have led to new challenges across multiple modalities of medical data.^5^^,^^6^ Facial images are at high risk given social media and openly available sources, especially with large public datasets used in facial recognition research.^5^^,^^7^ Furthermore, generative AI poses risks to conventional deidentification methods. Latent diffusion models support high-fidelity image synthesis and context-aware inpainting to reconstruct missing areas in images.^8^^,^^9^ Prior works have established that diffusion models achieve high image quality and allow for more control over the generation process.^10^^,^^11^ As summarized in Sordo et al.,^10^ diffusion models “achieve state-of-the-art image quality, often surpassing GANs and VAEs in realism and detail.” This raises important considerations for medical applications, particularly with the use of clinical photographs and patient privacy. Despite growing awareness of this risk, there is a lack of empirical evidence on the effectiveness of facial masking strategies in the context of modern AI capabilities. The community is asking: How much of a face is a face?

To address this gap, we apply an adversarial threat modeling framework.^12^ We leverage 10,000 high-resolution images from the Synthetic Faces High Quality (SFHQ) dataset^13^—a diverse and privacy-friendly alternative to real human face datasets^14^—as a large-scale, controlled simulation platform. We define specific facial regions of interest and evaluate the effectiveness of 14 regional masking strategies in mitigating reconstruction risk using a latent diffusion generative AI model.^9^ This work complements prior work on privacy assessment frameworks with the deidentification of full facial images.^15^^,^^16^ Unlike prior studies, we empirically assess how regional facial masking strategies affect generative AI reconstruction and face verification performance under this adversarial threat model. Specifically, this study (1) systematically evaluates conventional deidentification practices in the context of modern AI capabilities and (2) provides quantitative metrics of reidentification potential to inform deidentification standards and help define boundaries of safety for clinical images and patient privacy.

## 2 Methods

### 2.1 Data

This study uses the SFHQ dataset, which was selected for its high-resolution (1024×1024  pixels) and diversity across age, sex, and skin tone.^13^ Furthermore, synthetic datasets have emerged as privacy-friendly alternatives to real human face datasets.^17^^,^^18^ The SFHQ dataset comprises curated face images generated through a multi-step process. Briefly, Stable Diffusion v1.4 was used to produce initial images, which were refined by encoding them into the StyleGAN2 latent space with encoder4editing (e4e) and creating multiple high-quality candidates.^13^ Final selections for each image were made through a combination of human curation and automated filtering, ensuring realism and diversity across facial attributes and demographics.^13^

### 2.2 Regional Masking Strategies

**Fig. 1:**
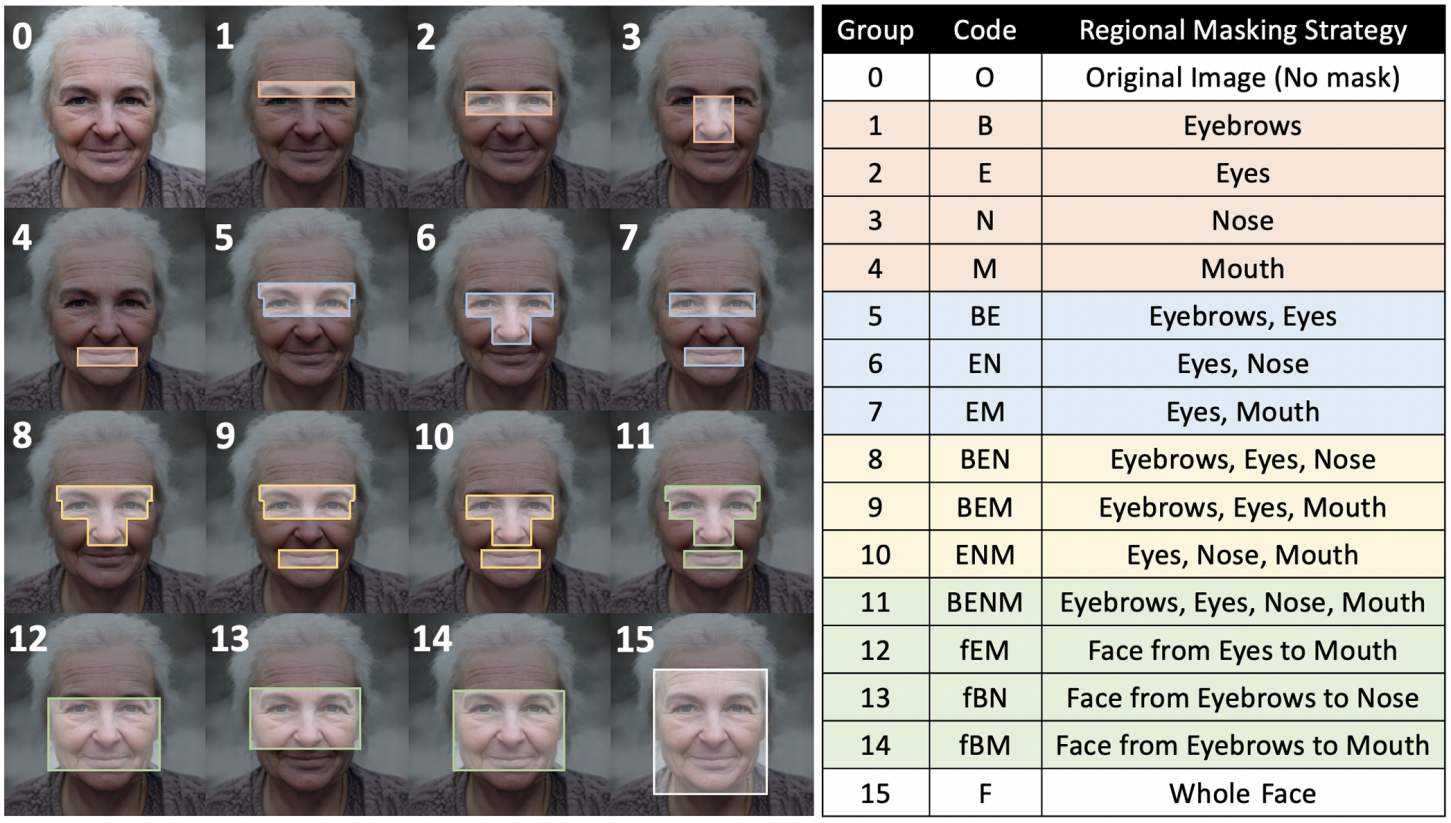
Regional masking strategies were defined based on anatomical facial regions of interest.

**Fig. 2:**
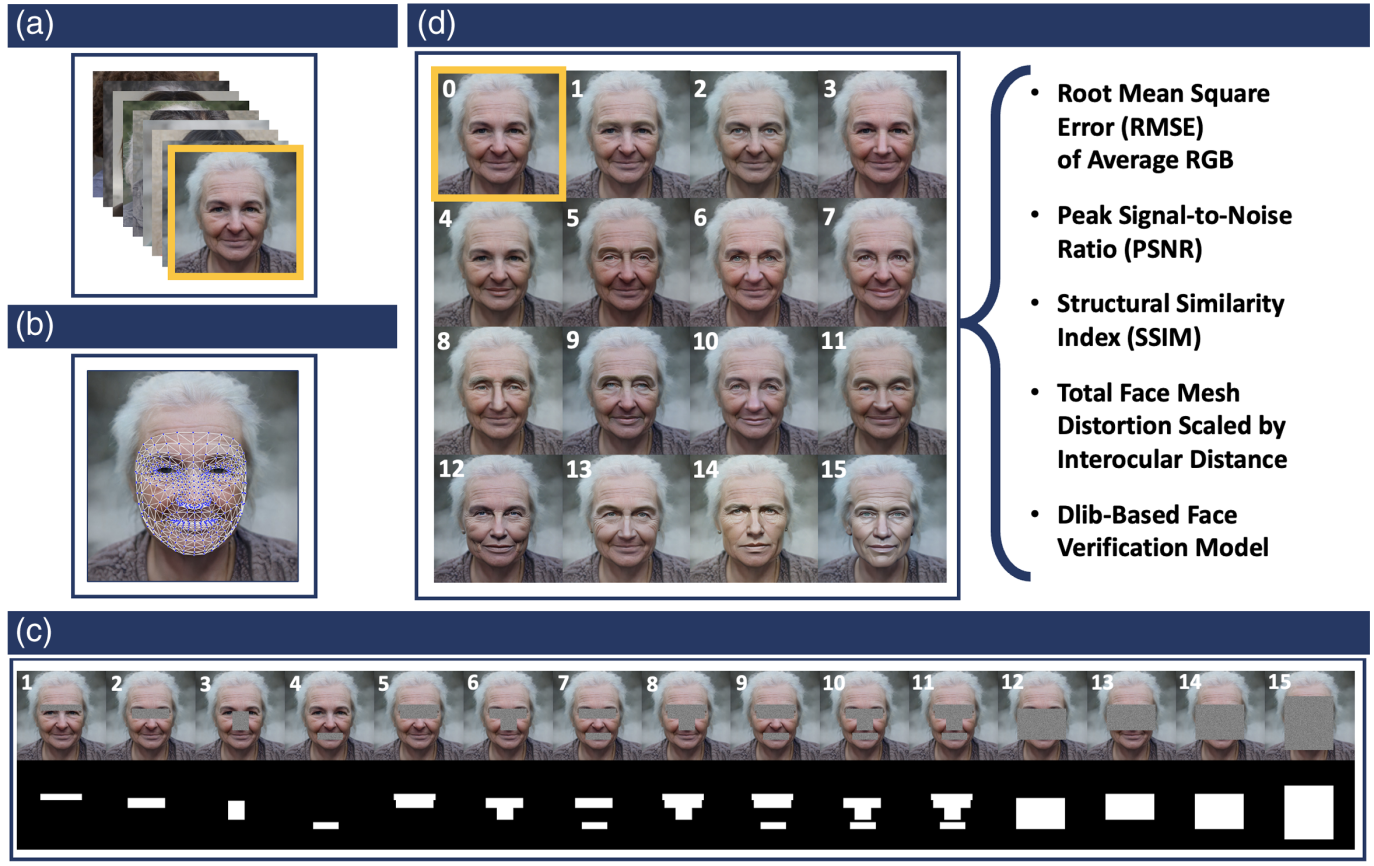
Overview of methodological pipeline. (a) Original images. We leverage 10,000 images from the SFHQ dataset as a large-scale simulation platform. (b) FaceMesh landmarks. Facial landmarks were mapped using the MediaPipe Face Mesh^19^ model. (c) Facial masking strategies and binary mask generation. Regions of interest were replaced by Gaussian noise to remove underlying facial information, and corresponding binary masks were generated for each region. (d) Generative AI reconstruction and analysis of reidentification potential. Generative AI reconstructions were performed with the SDXL Inpainting 0.1 latent diffusion model, using each preprocessed image-binary mask pair. Reidentification potential was assessed with quantitative image similarity metrics and an open-source face verification model.

Regional masking strategies were defined based on anatomical facial regions of interest (see Fig. 1. Facial landmarks were mapped using Google’s MediaPipe Face Mesh pipeline,^19^ which applies deep neural networks to extract 468 3D facial landmarks. The MediaPipe Face Mesh pipeline was run in static image mode to process each image independently. Landmarks were extracted in normalized coordinates and converted to pixel coordinates based on the input image resolution (1024×1024  pixels). The landmark indices used to localize specific facial features are specified in Table S4 in the Supplementary Material. For each region of interest, a tight bounding box was calculated by determining the minimum and maximum coordinates in the horizontal and vertical directions with a 10-pixel padding as shown in Fig. 1. Regions of interest were replaced by Gaussian noise to remove underlying facial information (see Fig. 2). For all images, Gaussian noise was drawn independently from a normal distribution with mean = 128 and standard deviation = 50, constrained to the valid pixel range [0, 255]. These parameters were selected to provide a neutral, random initialization that supports stable inpainting without meaningfully affecting comparative results, as the pixel values within masked regions are uninformative for the SDXL inpainting model, and the same parameters are applied uniformly across all masking strategies. Although the SDXL inpainting model accepts the original image and binary mask, we replaced the masked pixels directly in the original image prior to inpainting to ensure that information in these regions was not available to the model. The bounding boxes were also used to generate binary masks corresponding to each masking strategy (see Fig. 2). This enables region-specific evaluation of reidentification potential and supports quantitative, comparative analyses across masking strategies.

### 2.3 Generative AI Facial Reconstructions

Generative AI facial reconstructions were conducted with the SDXL 1.0 Inpainting version 0.1 latent diffusion model,^8^^,^^9^^,^^20^ using each preprocessed image-binary mask pair as inputs (see Fig. 2). The following parameters were used for inpainting: guidance scale = 10, inference steps = 50, strength = 0.99, and batch size = 1. Independent stochastic sampling was used to reflect realistic inpainting behavior and avoid bias from a fixed random seed. The DPMSolverMultistepScheduler^20^ was integrated to improve sampling efficiency and image quality through higher-order multistep inference. The experiments were run on an NVIDIA RTX A6000. We use the positive prompt: “Seamlessly fill in the image of a realistic human face with natural human facial features. Match realistic skin tone and texture in the surrounding image. Maintain photorealistic details and neutral lighting.” To suppress unwanted artifacts, we also specified negative prompts: “exaggerated, cartoon, anime, face mask, medical mask, text box, glare, artifacts, collage, sharp edges, unnatural colors, color blocks, color banding, textile, wrapping, animal print, distortion, asymmetrical, art, drawing, sketch, painting, costume, decoration, ornament, over-saturated, glassy eyes, cropped, out of frame, extra facial features, missing facial features, signature, words, letters, watermark, logo, low quality.” Manual quality assurance was performed, and images that failed to converge in any of the 15 groups were excluded from further analysis. Thus, 8702 images, with 130,530 total reconstructions across the 15 groups, were analyzed.

### 2.4 Analysis of Reidentification Potential

#### 2.4.1 Image similarity metrics

To assess pixel-level similarity between original and reconstructed facial images, we analyze the root mean square error (RMSE) of the average RGB values, peak signal-to-noise ratio (PSNR), and structural similarity index (SSIM). All metrics were calculated globally across full images and locally within masked regions of interest. SSIM was calculated using skimage.metrics.structural_similarity, which assesses perceptual similarity based on luminance, contrast, and structure across RGB channels. For local SSIM, a full-resolution similarity map was computed using the default 11×11 Gaussian-weighted window. SSIM values within the regions of interest were averaged to quantify local perceptual similarity.

#### 2.4.2 Geometric face mesh distortion

To quantify geometric changes, we calculated total face mesh distortion in normalized facial units. Google’s MediaPipe Face Mesh pipeline^19^ was applied to each image to extract the 468 3D facial landmarks. Euclidean displacements were computed between corresponding landmarks in the original and reconstructed images. These displacements were then scaled by interocular distance, providing a scale-invariant measure of geometric deformation across the face.

#### 2.4.3 Statistical analysis

**Fig. 3:**
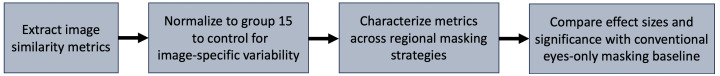
Overview outlining methods for comparative analyses.

For each image, metrics were normalized to group 15 (whole face) to control for image-specific variability (see Fig. 3). The median and IQR were calculated to characterize the distributions across groups. To compare the effectiveness of regional masking strategies, we performed pair-wise statistical comparisons between each group and group 2, which reflects the conventional eyes-only masking baseline. Effect sizes were quantified using paired Cohen’s d comparisons. Statistical significance was assessed using nonparametric two-tailed Wilcoxon signed-rank tests with an alpha level of 0.05. To account for multiple comparisons, p-values were adjusted using the Benjamini–Hochberg false discovery rate (FDR) correction.

#### 2.4.4 Face verification model

We applied an open-source, Dlib-based face verification model using the face_recognition Python library^21^^,^^22^. This deep learning model compares 128-dimensional face encodings based on facial structure, minimizing influence from background or hair style, to determine whether two images represent the same person. It has a reported accuracy of 99.38% on the Labeled Faces in the Wild (LFW) dataset, indicating that, when presented with a pair of face images, the model correctly classifies whether they belong to the same person 99.38% of the time.^22^^,^^23^ Thus, it is a strong, widely available face verification model, comparable with FaceNet (99.63% on LFW)^24^ and ArcFace (99.83% on LFW).^25^ In addition, it provides a stable, open-source, and well-established pipeline, making it a realistic choice for adversarial threat modeling. We kept all parameters at their default values (face detection model = “hog,” number_of_times_to_upsample = 1, landmark detection model = “large,” default ResNet-based face encoding model, and tolerance = 0.6). Although the tolerance parameter can affect the strictness of match decisions, tolerance = 0.6 is the standard, default threshold used by the face_recognition library to determine identity matches. These parameters were applied uniformly across all images. For each masking strategy, we calculated the percentage of images classified as matching the original image. We also calculated the 95% binomial confidence intervals using the Wilson score method. Relative performance was assessed by calculating the match rate ratio relative to group 2 (eyes only) and group 15 (whole face).

## 3 Results

### 3.1 Image Similarity Analyses

**Fig. 4:**
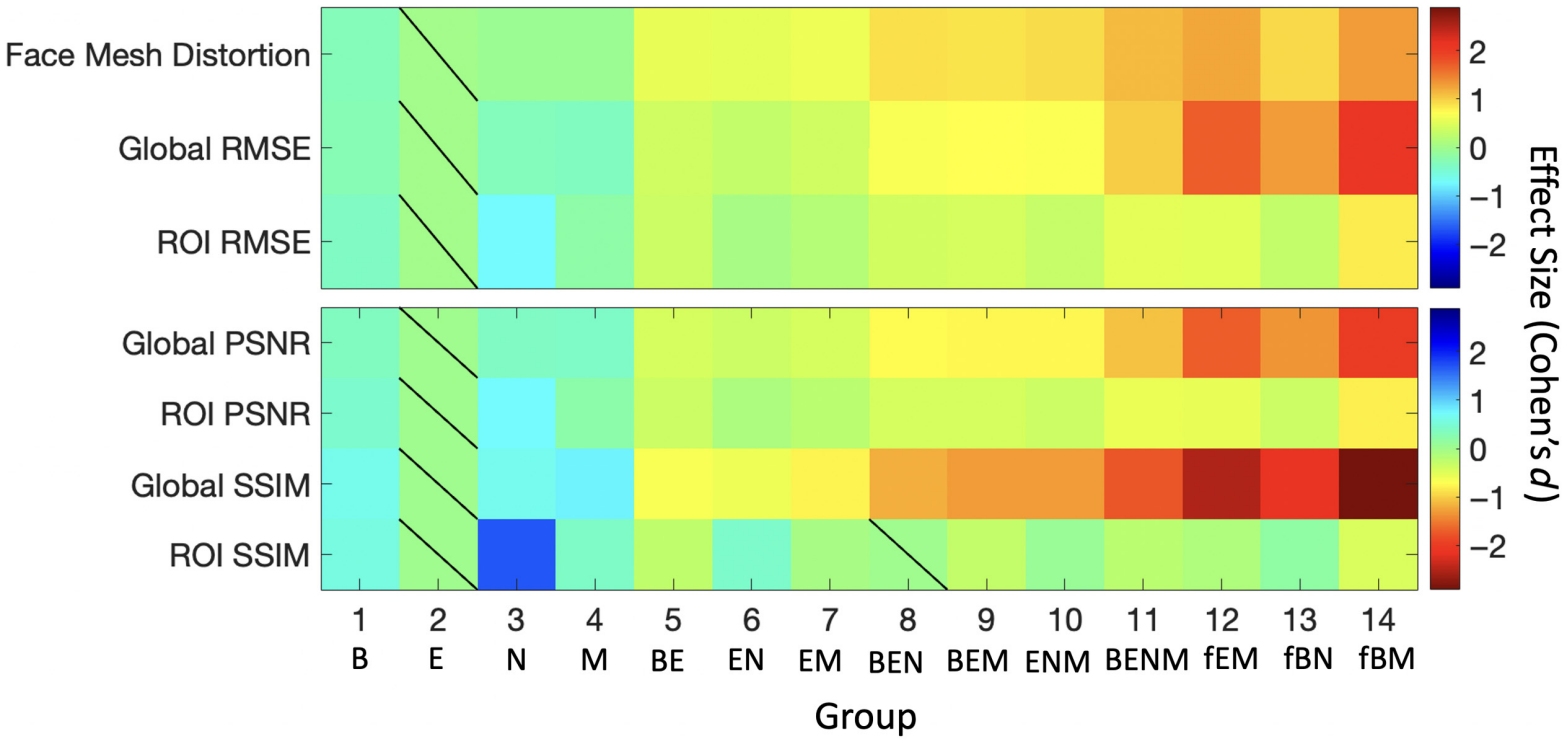
Masking all major facial features in group 11 achieves stronger protective effects compared with conventional eyes-only masking in group 2. This is reflected by large Cohen’s d effect sizes (d>1.0) across image similarity metrics of face mesh distortion and global RMSE, PSNR, and SSIM. Most ROI-based imaging metrics had |d|<0.6, suggesting consistent generative AI reconstruction quality across masking strategies. The color scale shows Cohen’s d effect sizes for paired comparisons of metric values normalized relative to group 15. Each masking strategy was compared with conventional eyes-only masking (group 2), and statistical significance was assessed with a two-tailed Wilcoxon signed-rank test with an alpha level of 0.05 after Benjamini–Hochberg FDR correction. Non-significant effects are indicated by a diagonal line. Stronger protective effects are reflected by higher face mesh distortion and RMSE values and by lower SSIM and PSNR values. The color scale is inverted for SSIM and PSNR to maintain consistent visual interpretations of “more protective” versus “less protective” across all metrics.

Image similarity between the original and reconstructed facial images was assessed using RMSE, PSNR, and SSIM metrics, normalized relative to group 15 (whole face). As shown in Fig. 4, generative AI reconstructions differed significantly across regional masking strategies. In particular, groups 11 to 14 had large Cohen’s d effect sizes (d>1.0) across image similarity metrics. Removing three facial features (groups 8 to 10) also provided enhanced protection, reflected by moderate effect sizes (d>0.7). Most local ROI changes remained comparable (|d|<0.6), suggesting consistent generative AI reconstruction quality across masking strategies. Notably, group 3 (nose only) had higher similarity to the original images with Cohen’s d effect sizes of 1.7 (ROI SSIM), 0.7 (ROI PSNR), and −0.7 (ROI RMSE). This may reflect its central location with more context from the surrounding face. Regardless of facial region, removing single features (groups 1 to 4), including conventional eyes-only masking, resulted in limited effectiveness. These quantitative analyses are qualitatively supported in Fig. 6.

### 3.2 Geometric Face Mesh Distortion

**Fig. 5:**
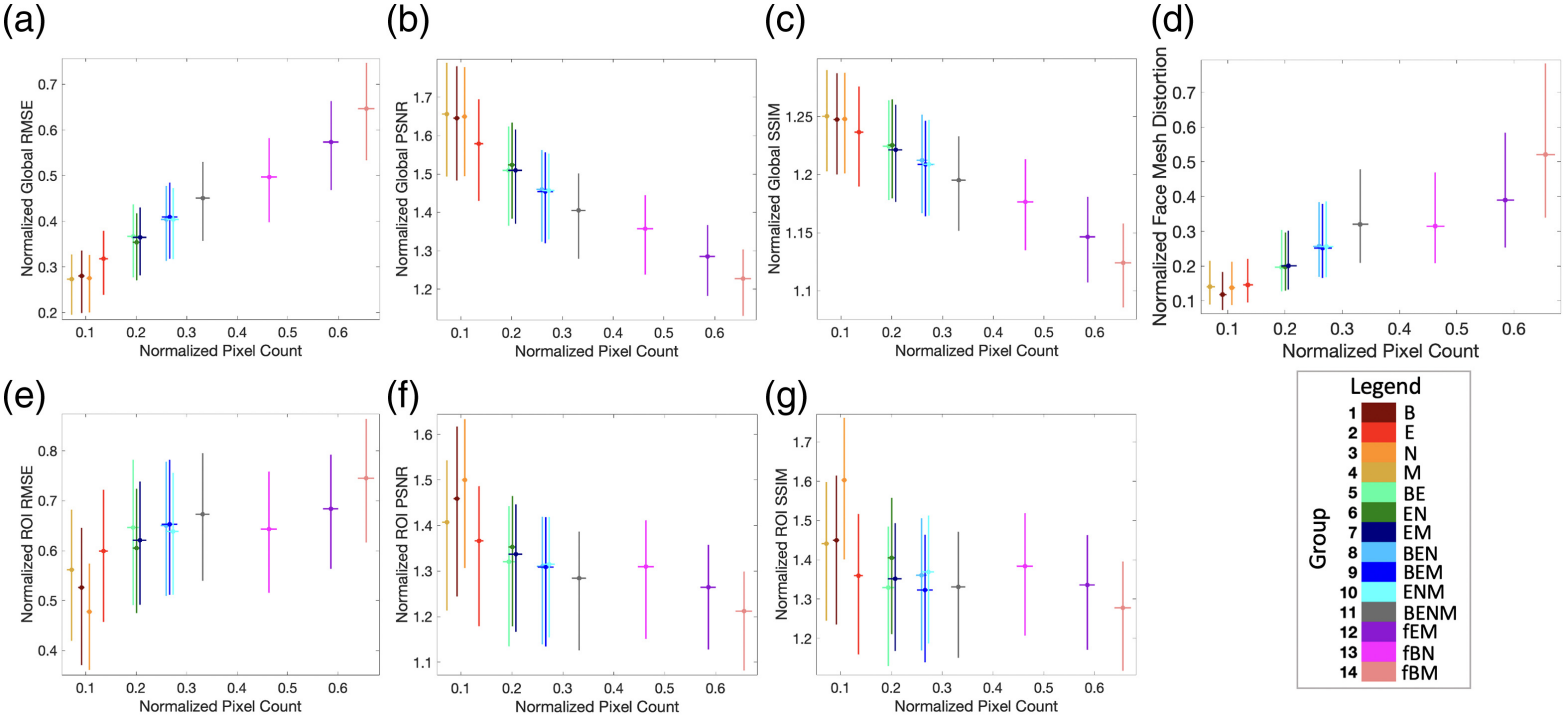
Quantitative distributions of image similarity metrics across regional masking strategies (median with IQR in the x and y axes). (a) Global RMSE. (b) Global PSNR. (c) Global SSIM. (d) Face mesh distortion. (e) ROI RMSE. (f) ROI PSNR. (g) ROI SSIM. All metrics were normalized relative to group 15. Absolute metric values are shown in Fig. S1 in the Supplementary Material. Regardless of facial region, single-feature changes (groups 1 to 4) resulted in comparable image similarity metrics, highlighting the limited effectiveness of masking individual regions in protecting identity.

To assess geometric changes in reconstructed faces, we calculated normalized face mesh distortion. As shown in Fig. 5(d), there were nonlinear relationships between face mesh distortion and mask area. Face mesh distortion remained low for all single-feature masks in groups 1 to 4, ranging from 0.14 to 0.18 relative to group 15, with absolute total face mesh distortions of 8.34 to 10.19 (see Tables S3 and S4 in the Supplementary Material). Regardless of facial region, these reconstructed images had minimal geometric deviations from the original face. Removing three facial features (groups 8 to 10) had moderate effects on total face mesh distortion with Cohen’s d>0.7. Groups 11 to 14 showed the most significant geometric changes, with Cohen’s d>1.0 relative to group 2 (see Fig. 4). However, expanding the masked area beyond group 11 did not consistently yield further gains. For example, group 13 produced only a marginal increase in total face mesh distortion despite masking larger areas of the face [see Table S1 in the Supplementary Material and Fig. 5(d)].

**Fig. 6:**
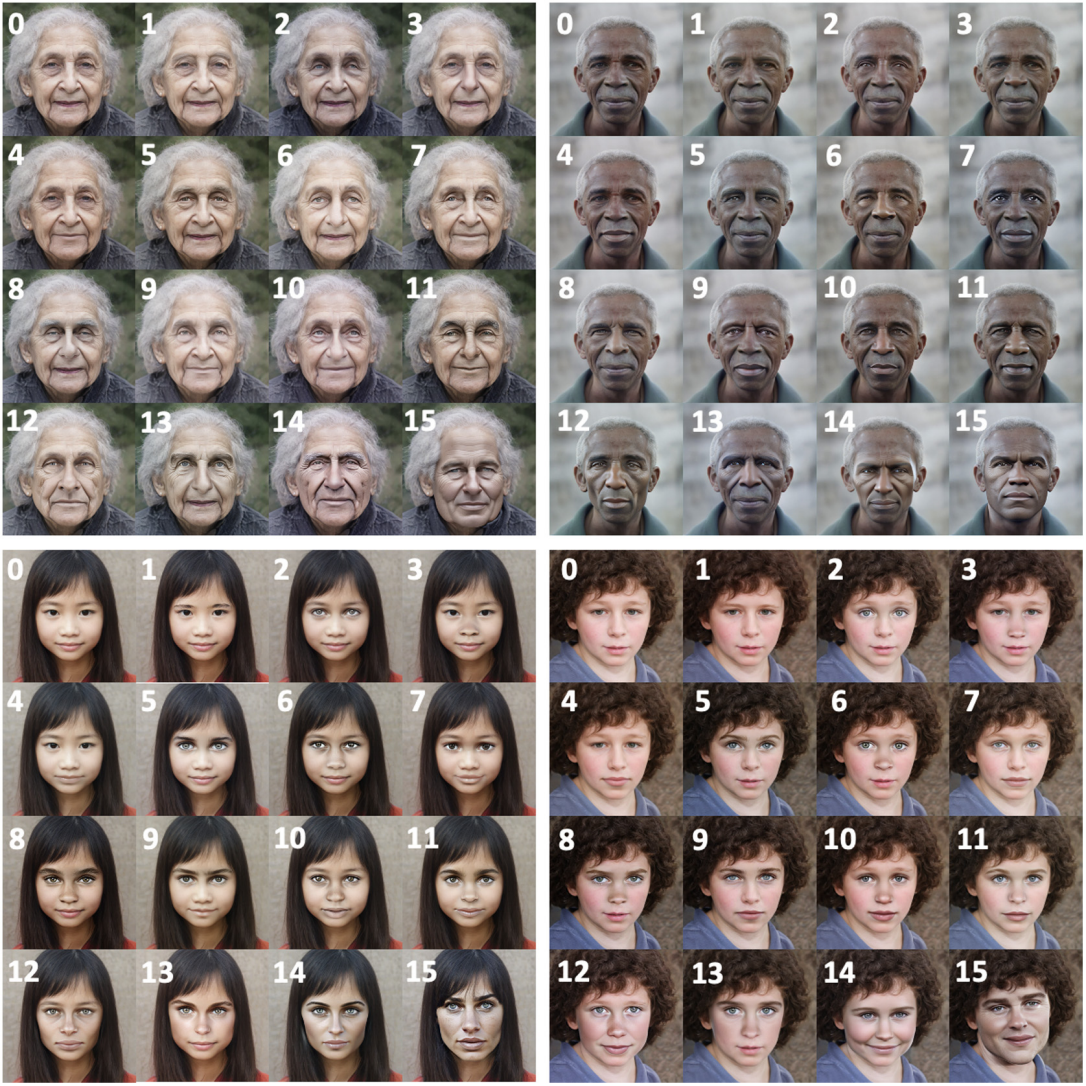
Example images qualitatively illustrate generative AI facial reconstructions across regional masking strategies. The visualizations demonstrate how the choice of masking strategy—such as occluding the eyes only (group 2) or all major facial features (group 11)—impacts the appearance and reidentification potential of generative AI reconstructions.

### 3.3 Effects on Face Verification Performance

**Fig. 7:**
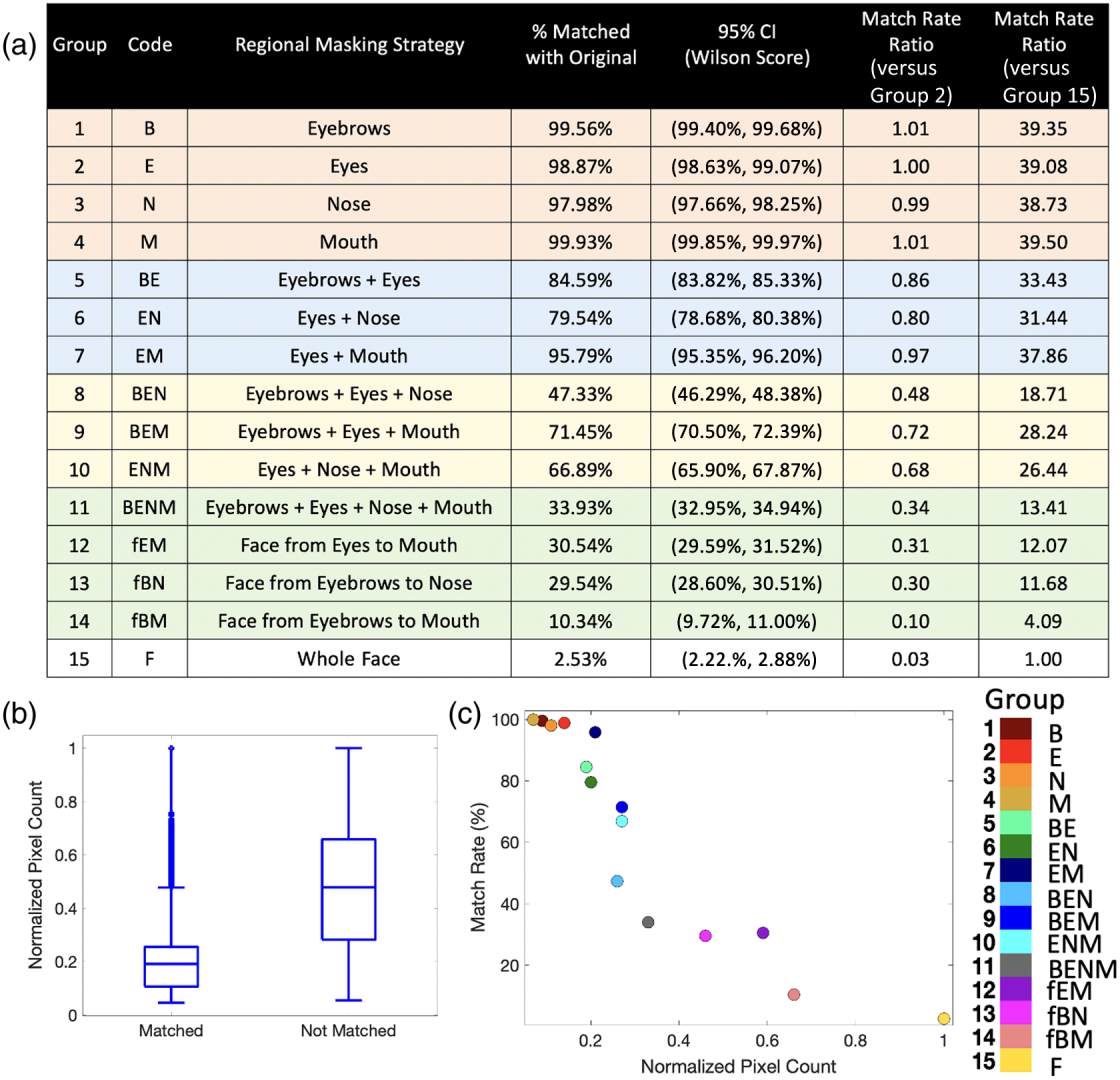
Regional masking strategies influence face verification performance. (a) 95% binomial confidence intervals were calculated using the Wilson score method. Relative performance was assessed by calculating the match rate ratio relative to group 2 (eyes only) and group 15 (whole face). These findings support the quantitative evaluations of image similarity metrics. (b) In general, face verification is less successful as more of the face is masked. (c) However, analysis across groups reveals important nonlinear patterns where masking all major facial features in group 11 was associated with significant reductions in face verification performance.

Regional masking strategies influenced face verification performance as demonstrated by the face verification match rates, Wilson score confidence intervals, and match rate ratios in Fig. 7(a). Single-feature masking (groups 1 to 4) resulted in high match rates ranging from 97.98% to 99.93%, consistent with the quantitative analyses across image similarity metrics. Removing three features had moderate decreases with match rates between 47.33% and 71.45%. Removing all major facial features (eyebrows, eyes, nose, and mouth) resulted in a threefold reduction in face verification rates compared with eyes alone, from 98.87% (95% CI [98.63%, 99.07%]) to 33.93% (95% CI [32.95%, 34.94%]). Figure S2 in the Supplementary Material illustrates example images that were matched and not matched across key groups. In general, the images were less likely to be reidentified as more of the face is masked [see Fig. 7(b)]. However, analysis across regional masking groups revealed important nonlinear patterns [see Fig. 7(c)]. In particular, masking the eyebrows, eyes, nose, and mouth together in group 11 was associated with significant reductions in reidentification potential. However, groups 12 and 13 had similar match rates to group 11, even though the masked area of group 12 was nearly double that of group 11 (see Table S1 in the Supplementary Material). Overall, these face verification findings further support the quantitative evaluations across image similarity and face mesh distortion metrics.

## 4 Discussion

### 4.1 Re-evaluating Conventional Deidentification Practices

Since the mid-20th century, clinical photographs have commonly been deidentified by placing black bars over the eyes to obscure a patient’s identity.^1^^,^^2^ Although increasingly questioned, this practice persists in clinical and academic settings amid a lack of standardized deidentification guidelines.^2^^–^^4^ With advancements in digital technologies to reconstruct facial images, it is essential to assess the reidentification potential associated with conventional facial masking strategies in the context of modern AI capabilities. We apply the SDXL 1.0 inpainting latent diffusion model to reconstruct facial images masked with black bars across combinations of facial features. When only the eyes were masked (group 2), the reconstructions exhibited high visual fidelity and structural coherence (see Figs. 5 and 6). The quantitative image similarity metrics were further supported by results from the face verification model, which correctly matched 98.87% of the reconstructed images to the original image (see Fig. 7). Taken together, these findings indicate that conventional deidentification methods of placing black bars over the eyes are insufficient and provide limited protection against AI-driven reconstruction. Furthermore, standardized deidentification guidelines must be informed empirically by the effectiveness of strategies in safeguarding against reidentification risk.

### 4.2 Effectiveness Across Regional Masking Strategies

Although the eyes have traditionally been seen as the most identifiable facial feature,^1^^,^^2^ we find that single feature changes resulted in similar levels of effectiveness regardless of facial region (see Fig. 5). Groups 1 to 4 demonstrated low face mesh distortion (0.14 to 0.18 relative to group 15; absolute total face mesh distortion 8.34 to 10.19) and high structural similarity index to the original face (1.24 to 1.25 relative to group 15; absolute SSIM 0.91 to 0.92) (see Tables S3 and S4 in the Supplementary Material). Correspondingly, face verification performance with single feature changes ranged from 97.98% to 99.93% (see Fig. 7). Overall, these results highlight the limited effectiveness of masking individual facial regions. In contrast, we find that masking core facial features together demonstrates marked improvements. Removing three facial features (groups 8 to 10) provided enhanced protection, reflected by moderate effect sizes (Cohen’s d>0.7) (see Fig. 4). Masking multiple facial features (group 11 to 14) achieved the strongest protective effects with Cohen’s d>1.0 across image similarity metrics (see Fig. 4). Interestingly, the relationship between reidentification potential and masked area was nonlinear (see Fig. 7). For example, removing all major facial features in group 11 reduced face verification rates to 33.93% (95% CI [32.95%, 34.94%]), compared with 98.87% (95% CI [98.63%, 99.07%]) in group 2. However, removing larger areas of the face did not consistently yield substantial gains. Notably, groups 12 and 13 were similar to group 11, even though the masked area of group 12 was nearly double that of group 11 (see Table S1 in the Supplementary Material). These patterns suggest a nonlinear threshold effect, where targeted masking of core facial features simultaneously disrupts anatomical coherence and mitigates accurate generative AI reconstructions. Overall, this systematic, region-specific evaluation provides an empirically grounded foundation to better inform safe deidentification standards.

### 4.3 Clinical Implications

This study highlights the potential reidentification risk that generative AI technologies pose for partially obscured faces. This raises important considerations for the medical community, particularly with clinical photographs and patient privacy. A central challenge in image deidentification is balancing privacy protection with the need to preserve clinically meaningful features. Our results demonstrate that simultaneously removing major facial features (eyebrows, eyes, nose, and mouth) significantly improves protection while still preserving large portions of the skin surface, which may be relevant for specialties such as dermatology. The optimal deidentification strategy is inherently dependent on the clinical application and patient population. Certain cases, such as disorders involving the ocular or periorbital regions, can involve features that are also highly identifying. Latent-space approaches are an emerging research area to disentangle anatomical and pathological features.^26^ In addition, populations with rare diseases or distinctive phenotypes may face elevated reidentification risk. In these cases where deidentification is not possible, informed consent procedures for publications or IRB protocols could include discussion of how images will be deidentified, the methods that are available, and their varying effectiveness. This would enable participants to make more informed decisions about acceptable levels of risk.

Our findings suggest that generative AI technologies can reduce the effectiveness of facial masking strategies under adversarial conditions. It is important to note that the risk to patient privacy is context-dependent.^27^ The highest-risk scenario involves public data, such as images in open repositories or datasets, particularly with AI and facial recognition research.^5^^,^^7^ Moderate-risk scenarios arise in user-controlled or social media environments where images are shared, but visibility varies by platform and user settings. Private clinical archives present lower risk contexts. In particular, cases where the original image is not publicly available or linked to an individual—for example, if a facial photo is taken in a clinic setting and is not externally accessible—may have more limited reidentification risk. Looking forward, developing empirical AI-aware deidentification standards can help define clearer boundaries for safe image use. This includes recognizing when traditional methods are acceptable and when more robust strategies are warranted. Incorporating these considerations may support safer and more consistent practices in using facial images across clinical and academic settings.

### 4.4 Limitations and Future Directions

It is important to acknowledge several limitations and future directions. First, our study uses the SFHQ dataset, which supports large-scale, controlled analyses and serves as a privacy-friendly alternative to real human face datasets.^14^^,^^17^^,^^18^ However, synthetic faces may exhibit fewer natural irregularities, which may underestimate reconstruction difficulty. In addition, they may not fully capture the complexity and variability of real-world conditions. The results apply primarily to front-facing, neutral-expression images and may vary for more oblique or expressive images. A valuable avenue for future work is to test robustness across natural variations, ideally with multiple photos per individual. This paper focuses on machine intelligence in reidentification potential, complementing other research areas such as human perception studies.^28^ Although we utilize the open-access SDXL 1.0 inpainting model and Dlib-based face verification model for adversarial threat modeling, future studies may explore other models. In addition, inpainting can be sensitive to prompt phrasing; although we defined a fixed prompt in this work, reconstruction realism may vary with alternative wording. Finally, our face verification analysis compares reconstructed images to the original image, which is less challenging than real-world scenarios with varied lighting, camera angles, and context. Nevertheless, evaluating strategies under strong threat models remains critical to inform safe deidentification standards.

## 5 Conclusion

This study presents a systematic evaluation of conventional deidentification practices in the context of modern AI capabilities and provides quantitative metrics of reidentification potential to inform deidentification standards and help define boundaries of safety for clinical images and patient privacy. Our findings demonstrate that the long-standing clinical practice of placing black bars over the eyes provides limited protection against generative AI facial reconstruction. We found that removing major facial features (eyebrows, eyes, nose, and mouth) simultaneously significantly improves protection while preserving large portions of the skin surface, which may be useful for specialties such as dermatology. This work underscores the importance of empirical, AI-aware deidentification standards that adapt to evolving technological capabilities. This is particularly relevant in medical contexts where patient privacy is a primary concern and where facial images are shared, published, or archived across clinical and academic settings.

## Supplementary Material

10.1117/1.JMI.13.S1. S11202.s01

## Data Availability

The dataset used in this study is available at https://doi.org/10.34740/kaggle/dsv/4737578. Supplementary materials are available: Fig. S1 in the Supplementary Material shows quantitative distributions of image similarity metrics across regional masking strategies. Figure S2 in the Supplementary Material shows example images illustrating face verification outcomes for original and reconstructed images across key groups. Tables S1–S3 in the Supplementary Material provide raw and normalized pixel counts and image similarity scores across masking strategies. Table S4 in the Supplementary Material provides face mesh landmark indices used to define regions of interest.
