# Supplementary material for: How much of a face is a face: exploring reidentification potential with generative AI
Source: J Med Imaging (Bellingham). 2026 Feb 15;13(Suppl 1):S11202. doi: 10.1117/1.JMI.13.S1.S11202 (PMC12906867; doi:10.1117/1.JMI.13.S1.S11202)
Supplement: Supplementary file 1 [file JMI_013_S11202_SD001.pdf]

Supplementary Materials

"How much of a face is a face: Exploring reidentification potential with generative AI"

Chloe Cho, Yihao Liu, Bohan Jiang, Andrew J. McNeil, Benoit M. Dawant,
Bennett A. Landman, Eric R. Tkaczyk

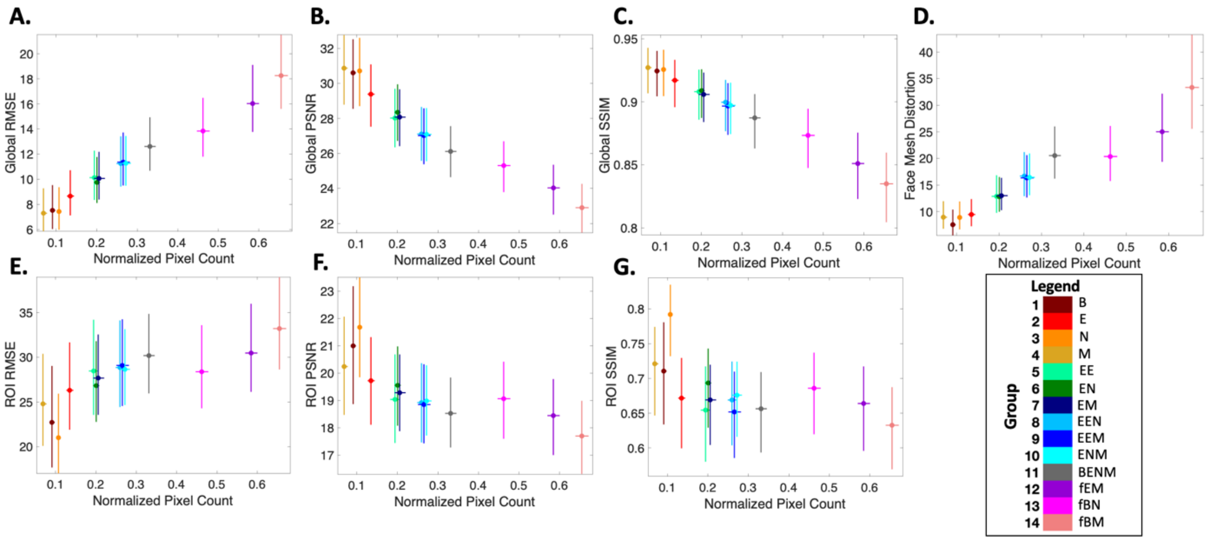

Supplementary Fig. S1 Quantitative distributions of image similarity metrics across regional masking strategies (median with IQR in the x and y axes): A) Global RMSE, B) Global PSNR, C) Global SSIM, D) Face Mesh Distortion, E) ROI RMSE, F) ROI PSNR, G) ROI SSIM.

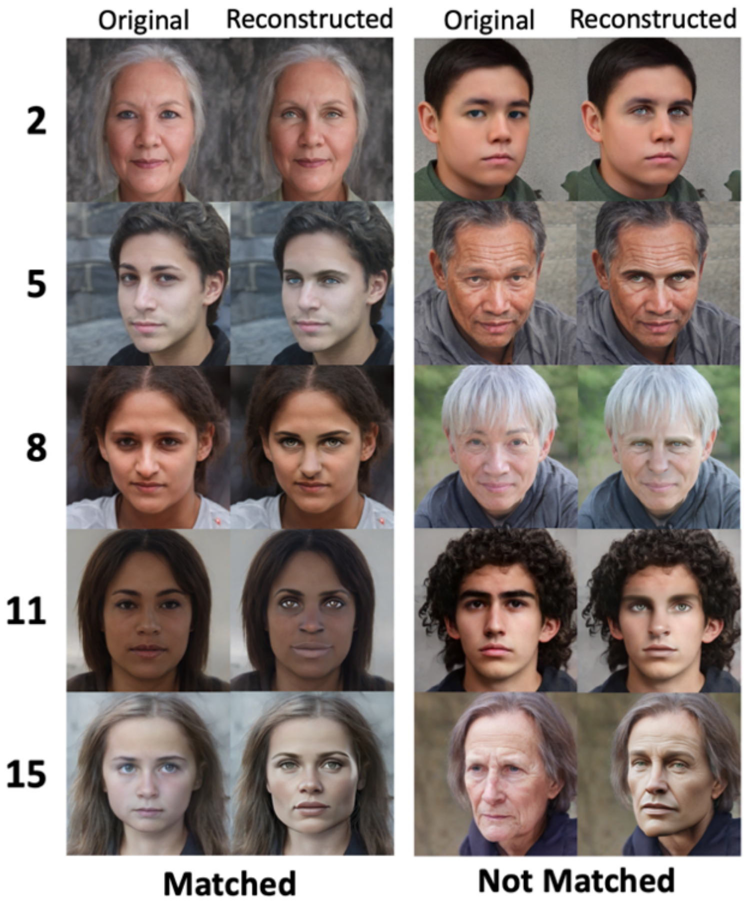

Supplementary Fig. S2 Example images illustrate face verification performance with original and reconstructed images that were matched and not matched across key groups.

Supplementary Table S1 Pixel Count (mean ± stdev) For Each Masking Strategy

Group	Code	Masking Strategy	Masked Area (pixels)	Normalized Masked Area
1	B	Eyebrows	35106 ± 4285	0.09 ± 0.01
2	E	Eyes	51598 ± 5282	0.14 ± 0.01
3	N	Nose	40993 ± 3819	0.11 ± 0.01
4	M	Mouth	27603 ± 5980	0.07 ± 0.01
5	BE	Eyebrows + Eyes	73952 ± 7201	0.19 ± 0.02
6	EN	Eyes + Nose	76570 ± 5990	0.20 ± 0.01
7	EM	Eyes + Mouth	79201 ± 9097	0.21 ± 0.02
8	BEN	Eyebrows + Eyes + Nose	98924 ± 7992	0.26 ± 0.02
9	BEM	Eyebrows + Eyes + Mouth	101555 ± 10393	0.27 ± 0.02
10	ENM	Eyes + Nose + Mouth	104169 ± 9718	0.27 ± 0.02
11	BENM	Eyebrows + Eyes + Nose + Mouth	126523 ± 11124	0.33 ± 0.02
12	fEM	Face from Eyes to Mouth	223550 ± 19282	0.59 ± 0.02
13	fBN	Face from Eyebrows to Nose	176767 ± 16443	0.46 ± 0.02
14	fBM	Face from Eyebrows to Mouth	250256 ± 21476	0.66 ± 0.02
15	F	Whole-Face	381899 ± 32617	1.00 ± 0.00

Supplementary Table S2 Raw Image Similarity Scores (mean ± stdev)

Group	Masking Strategy	Face Mesh Distortion (normalized facial units)	RMSE of Average RGB		PSNR		SSIM	
			Global	ROI	Global	ROI	Global	ROI
1	B	8.34 ± 3.84	8.05 ± 2.80	24.13 ± 8.81	30.51 ± 2.90	21.04 ± 3.12	0.92 ± 0.03	0.70 ± 0.11
2	E	10.19 ± 4.10	9.16 ± 2.80	27.43 ± 7.64	29.28 ± 2.55	19.68 ± 2.34	0.91 ± 0.03	0.66 ± 0.10
3	N	9.79 ± 4.47	7.94 ± 2.69	22.03 ± 6.77	30.59 ± 2.79	21.66 ± 2.61	0.92 ± 0.03	0.77 ± 0.08
4	M	9.79 ± 4.24	7.86 ± 2.74	25.78 ± 7.85	30.70 ± 2.85	20.30 ± 2.63	0.92 ± 0.03	0.70 ± 0.11
5	BE	13.76 ± 5.43	10.58 ± 3.06	29.60 ± 8.33	27.99 ± 2.44	19.03 ± 2.38	0.90 ± 0.03	0.64 ± 0.10
6	EN	13.69 ± 5.24	10.22 ± 2.87	27.79 ± 7.00	28.26 ± 2.35	19.52 ± 2.13	0.90 ± 0.03	0.68 ± 0.09
7	EM	13.78 ± 4.89	10.53 ± 2.96	28.49 ± 6.93	28.00 ± 2.35	19.29 ± 2.07	0.90 ± 0.03	0.65 ± 0.09
8	BEN	17.59 ± 6.48	11.67 ± 3.10	29.83 ± 7.46	27.08 ± 2.25	18.90 ± 2.12	0.89 ± 0.04	0.66 ± 0.09
9	BEM	17.15 ± 6.06	11.82 ± 3.18	29.93 ± 7.45	26.98 ± 2.29	18.87 ± 2.13	0.89 ± 0.04	0.64 ± 0.10
10	ENM	17.53 ± 6.28	11.70 ± 3.03	29.37 ± 6.56	27.04 ± 2.18	18.98 ± 1.91	0.89 ± 0.04	0.66 ± 0.09
11	BENM	21.68 ± 7.60	13.05 ± 3.28	30.93 ± 7.11	26.08 ± 2.12	18.54 ± 1.94	0.88 ± 0.04	0.64 ± 0.09
12	fEM	26.79 ± 10.41	16.76 ± 4.23	31.82 ± 8.07	23.90 ± 2.09	18.33 ± 2.05	0.85 ± 0.04	0.65 ± 0.09
13	fBN	21.66 ± 9.10	14.44 ± 3.75	29.74 ± 7.87	25.21 ± 2.15	18.93 ± 2.12	0.87 ± 0.04	0.67 ± 0.09
14	fBM	36.03 ± 14.85	18.92 ± 4.63	34.68 ± 8.76	22.83 ± 2.02	17.57 ± 2.02	0.83 ± 0.04	0.62 ± 0.09
15	F	73.44 ± 37.64	30.23 ± 7.31	48.18 ± 12.00	18.76 ± 2.02	14.72 ± 2.07	0.74 ± 0.05	0.50 ± 0.09

Supplementary Table S3 Normalized Image Similarity Scores (mean ± stdev)

Group	Masking Strategy	Face Mesh Distortion (normalized facial units)	RMSE of Average RGB		PSNR		SSIM	
			Global	ROI	Global	ROI	Global	ROI
1	B	0.14 ± 0.10	0.28 ± 0.12	0.53 ± 0.22	1.65 ± 0.24	1.46 ± 0.32	1.25 ± 0.07	1.45 ± 0.30
2	E	0.18 ± 0.12	0.32 ± 0.12	0.60 ± 0.20	1.58 ± 0.23	1.37 ± 0.28	1.24 ± 0.07	1.36 ± 0.28
3	N	0.17 ± 0.12	0.28 ± 0.11	0.48 ± 0.16	1.65 ± 0.23	1.50 ± 0.29	1.25 ± 0.07	1.60 ± 0.29
4	M	0.17 ± 0.11	0.27 ± 0.11	0.56 ± 0.20	1.66 ± 0.24	1.41 ± 0.29	1.25 ± 0.07	1.44 ± 0.29
5	BE	0.24 ± 0.15	0.37 ± 0.13	0.65 ± 0.22	1.51 ± 0.21	1.32 ± 0.27	1.22 ± 0.06	1.33 ± 0.28
6	EN	0.24 ± 0.16	0.35 ± 0.12	0.61 ± 0.18	1.52 ± 0.21	1.35 ± 0.26	1.23 ± 0.06	1.41 ± 0.27
7	EM	0.24 ± 0.15	0.36 ± 0.12	0.62 ± 0.19	1.51 ± 0.21	1.34 ± 0.25	1.22 ± 0.06	1.35 ± 0.26
8	BEN	0.30 ± 0.19	0.40 ± 0.13	0.65 ± 0.20	1.46 ± 0.20	1.31 ± 0.25	1.21 ± 0.06	1.36 ± 0.27
9	BEM	0.29 ± 0.18	0.41 ± 0.13	0.65 ± 0.20	1.45 ± 0.20	1.31 ± 0.26	1.21 ± 0.06	1.32 ± 0.26
10	ENM	0.30 ± 0.19	0.40 ± 0.12	0.64 ± 0.18	1.46 ± 0.19	1.31 ± 0.24	1.21 ± 0.06	1.37 ± 0.25
11	BENM	0.37 ± 0.23	0.45 ± 0.13	0.67 ± 0.19	1.41 ± 0.18	1.28 ± 0.24	1.20 ± 0.06	1.33 ± 0.25
12	fEM	0.46 ± 0.28	0.57 ± 0.15	0.68 ± 0.18	1.29 ± 0.15	1.26 ± 0.20	1.15 ± 0.06	1.34 ± 0.24
13	fBN	0.37 ± 0.25	0.50 ± 0.14	0.64 ± 0.18	1.36 ± 0.18	1.31 ± 0.24	1.18 ± 0.06	1.38 ± 0.25
14	fBM	0.61 ± 0.39	0.65 ± 0.16	0.75 ± 0.19	1.23 ± 0.14	1.21 ± 0.20	1.12 ± 0.06	1.28 ± 0.23
15	F	1.00 ± 0.00	1.00 ± 0.00	1.00 ± 0.00	1.00 ± 0.00	1.00 ± 0.00	1.00 ± 0.00	1.00 ± 0.00

Supplementary Table S4 Face Mesh Landmarks for Regions of Interest

Facial Feature	MediaPipe Face Mesh Landmarks
Right eye	{226, 31, 228, 229, 230, 231, 232, 233, 244, 189, 221, 222, 223, 224, 225, 113}
Left eye	{446, 342, 445, 444, 443, 442, 441, 413, 464, 453, 452, 451, 450, 449, 448, 261}
Right eyebrow	{70, 63, 105, 66, 107, 55, 65, 52, 53, 46}
Left eyebrow	{336, 296, 334, 293, 300, 276, 283, 282, 295, 285}
Nose	{168, 193, 245, 188, 174, 198, 49, 102, 64, 240, 97, 2, 326, 460, 294, 331, 279, 420, 399, 412, 465, 417}
Mouth	{57, 185, 40, 39, 37, 0, 267, 269, 270, 409, 287, 375, 321, 405, 314, 17, 84, 181, 91, 146}
Face from Eyes to Mouth	{168, 417, 441, 442, 443, 444, 445, 342, 446, 265, 372, 264, 356, 454, 323, 401, 433, 416, 434, 432, 287, 375, 321, 405, 314, 17, 84, 181, 91, 146, 57, 212, 214, 192, 213, 177, 93, 234, 127, 34, 143, 35, 226, 113, 225, 224, 223, 222, 221, 193}
Face from Eyebrows to Nose	{9, 336, 296, 334, 293, 300, 383, 372, 264, 356, 454, 323, 366, 352, 280, 425, 266, 423, 327, 326, 2, 97, 98, 203, 36, 205, 50, 123, 137, 93, 234, 127, 34, 143, 156, 70, 63, 105, 66, 107}
Face from Eyebrows to Mouth	{9, 336, 296, 334, 293, 300, 383, 372, 264, 356, 454, 323, 401, 433, 416, 434, 432, 287, 375, 321, 405, 314, 17, 84, 181, 91, 146, 57, 212, 214, 192, 213, 177, 93, 234, 127, 34, 143, 156, 70, 63, 105, 66, 107}
Whole-Face	{10, 338, 297, 332, 284, 251, 389, 356, 454, 323, 361, 288, 397, 365, 379, 378, 400, 377, 152, 148, 176, 149, 150, 136, 172, 58, 132, 93, 234, 127, 162, 21, 54, 103, 67, 109}

*MediaPipe FaceMesh Landmark Indices Reference: https://github.com/google-ai-edge/mediapipe/blob/63e679d99ca45b30514a9d84c9351a2d77bb9ba0/mediapipe/modules/face_geometry/data/canonical_face_model_uv_visualization.png
